# Longitudinal changes of self-perceived manual ability the first year after stroke: a cohort study

**DOI:** 10.1186/s12883-020-01754-9

**Published:** 2020-05-12

**Authors:** Elisabeth Ekstrand, Katharina S. Sunnerhagen, Hanna C. Persson, Åsa Lundgren-Nilsson, Margit Alt Murphy

**Affiliations:** 1 Department of Health Sciences, Lund University, Lund, Sweden; 2 Department of Hand Surgery, Skåne University Hospital, Malmö, Sweden; 3 Department of Clinical Neuroscience, Rehabilitation Medicine, Institute of Neuroscience and Physiology, Sahlgrenska Academy, University of Gothenburg, Per Dubbsgatan 14, 3tr, S-413 45 Göteborg, Sweden

**Keywords:** Activities of daily living, Longitudinal study, Recovery, Stroke, Self report, Upper extremity

## Abstract

**Background:**

Recovery patterns of motor function and activity capacity of the upper extremity after stroke have been described, but less is known about longitudinal changes of perceived manual activity performance. The aim of this study was to investigate longitudinal changes of self-perceived manual ability at several timepoints from onset until 12 months post-stroke in a cohort of consecutively recruited individuals with mild, moderate and severe stroke.

**Methods:**

The study included 106 participants from a non-selected cohort with first-ever mild, moderate or severe stroke and impaired upper extremity function (Stroke Arm Longitudinal Study at the University of Gothenburg, SALGOT). Self-perceived manual ability was assessed with the ABILHAND Questionnaire at 3 and 10 days, 4 weeks, 3, 6 and 12 months after stroke. Longitudinal change was assessed by linear mixed models (fixed and random effects) and adjusted models were built by adding effects of cofactors age, gender, stroke severity, living condition and affected hand.

**Results:**

Self-perceived manual ability increased over time the first year after stroke for the total group and the subgroups. The final adjusted model for the total group included fix-effects of time (expected mean change 0.24 logits per month) adjusted by age (− 0.06 per year) and stroke severity (− 0.19 per NIHSS-score). In addition to significant effect of time, the adjusted models for moderate stroke subgroup included fixed effect of age, and for mild and severe subgroups there was an interaction effect between time and age. Further analyses between time-points showed that no significant change of self-perceived manual ability was detected beyond 3 months post-stroke.

**Conclusions:**

Self-perceived manual ability increased over time the first year after stroke, and this change was to some degree modulated by age and stroke severity at onset. Most of the improvements occurred early, predominantly within the first three months after stroke.

## Background

After stroke, impairments of the upper extremity are common and affect approximately 50–70% of the stroke survivors in the acute stage [1, 2] and 40% in the chronic phase [1, 3]. A remaining upper extremity impairment may negatively impact daily life and limit the ability to perform manual activities necessary for independent living [4]. To regain manual ability is therefore an important goal in stroke rehabilitation [5].

To follow recovery and effects of interventions after stroke different aspects of the ICF (International Classification of Functioning, Disability and Health) [4] needs to be considered. There are recommendations of core measures after stroke [6] to be used such as the National Institutes of Health Stroke Scale (NIHSS) [7] to measure stroke severity, the Fugl-Meyer Assessment for Upper Extremity (FMA-UE) [8] to measure upper extremity impairments and Action Research Arm Test (ARAT) [9] to measure activity capacity limitations. As recovery of functions do not automatically lead to improvements in performing upper extremity activities in real life [10] measures of activity performance are also essential to include. Self-reported activity measures, such as the ABILHAND Questionnaire [11], provide important information of the performance of activities in daily life after stroke that is relevant from the perspective of the person [12].

After a stroke, the major part of recovery of the upper extremity usually takes place within the first few months but improvements can continue for a longer time [10]. Regained body functions are mainly due to spontaneous neurobiological recovery [13] while improvement of activities depends on complex interaction between upper extremity functions and adaptive and compensatory behaviours [14]. Recovery has been found to differ depending on stroke severity, being more predictable for individuals with mild and moderate stroke compared to severe strokes [15]. Assessments of upper extremity motor function and activity capacity has been shown to be equally effective to measure recovery in the acute and early subacute stage post-stroke [16], but less is known about longitudinal changes of perceived manual activity performance. In previous studies increased self-reported amount of use of the upper extremity has been reported between discharge from inpatient rehabilitation and 12 months after stroke [17]. Increased perceived manual ability (ABILHAND logits) has also been demonstrated in a longitudinal study with several follow-up time-points in the rehabilitation and post-rehabilitation phase [18]. Both studies included, however, participants at average of 1 month after stroke onset and used follow-up time-points that varied according to the participant’s admission and/or discharge times [17, 18]. Thus, studies are lacking that investigate longitudinal changes of perceived activity performance by repeated measurements in well-defined time-points from stroke onset well into the chronic phase. Increased understanding about perceived manual ability after stroke would facilitate efficient goal setting and individualised rehabilitation interventions with appropriate timing after stroke.

The aim of this study was to investigate longitudinal changes of self-perceived manual ability at several time-points from onset until 12 months post-stroke in a cohort of consecutively recruited individuals with mild, moderate and severe stroke.

## Methods

### Participants

**Fig. 1:**
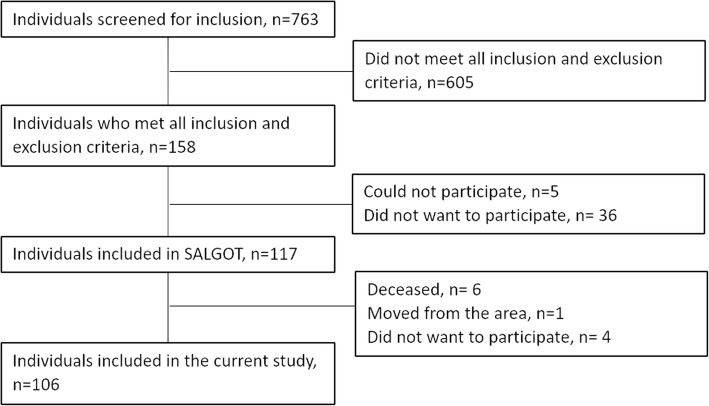
Flow chart of inclusion

This longitudinal study is a part of the Stroke Arm Longitudinal Study at the University of Gothenburg (SALGOT). The SALGOT-study comprised a non-selected cohort of 117 adults with first-ever stroke admitted to the largest stroke unit at Sahlgrenska University Hospital in Sweden during a 18 month period between 2009 to 2010 [19]. Inclusion criteria were i) ischemic or haemorrhagic stroke (confirmed by clinical neuroimaging); ii) upper extremity disability at 3 days after stroke onset (Action Research Arm Test, ARAT < 57); iii) resident in Gothenburg urban area; iv) 18 years or older; v) Swedish speaking; vi) no other upper extremity condition that limited the functional use of arm and hand; vii) no severe multi-impairment, diminished physical condition or short life expectancy due to other chronic or terminal illness prior to stroke. The current study included data from 106 participants that responded to the ABILHAND Questionnaire (self-perceived manual ability) at 3 and 10 days, 4 weeks, 3, 6 or 12 months after stroke [19] (see Fig. 1). All participants received individually adjusted task-specific rehabilitation (physiotherapy and/or occupational therapy) from stroke onset (first day at stroke unit). Continued rehabilitation (in-patient and/or outpatient) was given based on the individuals’ needs according to the Swedish national guidelines [20].

### Assessment

Stroke severity at admission was measured by the NIHSS [7]. The total score ranges from 0 (no deficit) to 42 (severe deficit) [21]. Based on the NIHSS score the participants were allocated to one of three subgroups; mild (NIHSS score < 5), moderate (NIHSS score 5 to 14) and severe stroke (NIHSS score > 14) [7]. Barrow Neurological Institute (BNI) [22] pre-screening, with a maximum score of 9 points, was performed to assess level of alertness, basal communication and co-operation in order to verify that the persons could adequately take part in assessments and respond to self-reported measures. Upper extremity motor and sensory function 3 days post stroke were assessed by the FMA-UE [8] and grip strength, calculated as percentage of reference values adjusted for age, sex and hand dominance, by the Jamar dynamometer (Sammons Preston, Chicago) [23, 24].

Self-perceived manual ability was assessed by the ABILHAND Questionnaire [11], Table 1. The ABILHAND is validated, has acceptable test-retest reliability and is recommended for assessment of upper extremity activity performance for individuals with stroke [11, 25, 26]. It includes 23 common bimanual tasks that are rated during an interview as impossible, difficult or easy. By the Rasch measurement model the score is converted into an interval scale in logits (i.e. log odds units) distributed around zero which indicates the centre of the scale [27]. A higher positive logit score indicates better self-perceived manual ability. If a participant could not answer to the questions of the ABILHAND, the test was recorded as missing.

**Table 1:** The ABILHAND Questionnaire

Items	
1	Hammering a nail
2	Threading a needle
3	Peeling potatoes
4	Cutting one’s nails
5	Wrapping up gifts
6	Cutting meat
7	Filing one’s nails
8	Peeling onions
9	Shelling hazel nuts
10	Opening a screw-topped jar
11	Fastening the zipper of a jacket
12	Tearing open a pack of chips
13	Buttoning up a shirt
14	Sharpening a pencil
15	Taking the cap off a bottle
16	Spreading butter on a slice of bread
17	Fastening a snap (jacket, bag)
18	Buttoning up trousers
19	Opening mail
20	Pulling up the zipper of trousers
21	Squeezing tooth-paste on a toothbrush
22	Unwrapping a chocolate bar
23	Washing one’s hands

Items ranked from 1 to 23 are ordered from more difficult to less difficult

### Statistics

Descriptive statistics of frequencies, means, standard deviations (SD), medians and interquartile ranges were used for clinical and demographic characteristics.

Longitudinal change of self-perceived manual ability was assessed by linear mixed models. Model assumptions were checked by means of residual analysis. Linear mixed models take into account missing values and that observations are not independent (several measurements per individual). The models were tested for different levels of fixed and random effects in order to see if more variance could be explained by separate effects. Adjusted models were built by adding effects of the cofactors age, gender, stroke severity (NIHSS-score), living condition (living alone) and affected hand (dominant). Interaction effects between cofactors were also analysed. The maximum likelihood method was used to determine the significance of each new model.

Boxplots were used to visually display self-perceived manual ability (ABILHAND logits) over time and differences in mean scores between time-points were evaluated by Friedman’s two-way analysis of variance by ranks.

Data were analysed with the IBM SPSS Statistics version 25 (IBM Corporation, Armonk, New York, United States). Probability values less than 0.05 were defined as statistically significant. Significance values were adjusted by the Bonferroni correction for multiple tests.

## Results

### Participants

Table 2 presents the demographic and clinical characteristics of the 106 participants and subgroups of mild (*n* = 41), moderate (*n* = 46) and severe stroke (*n* = 19). The mean age of the total group was 68 years (SD 13) and individuals in the subgroup mild were slightly younger. The dominant hand was affected in 43% of the individuals and the majority (90%) had lower grip strength in the more affected hand compared to normative values.

**Table 2:** Demographic and clinical characteristics at stroke onset

	Total group	Mild	Moderate	Severe
Individuals per group, n (%)	106 (100)	41 (39)	46 (43)	19 (18)
Age, mean years (SD; min-max)	68 (13; 26–95)	66 (14; 26–89)	69 (11; 38–90	71 (14; 34–95)
Gender (male), n (%)	61 (57.5)	23 (56)	25 (54)	13 (68)
Living situation (living alone), n (%)	41 (39)	20 (49)	17 (37)	4 (21)
Affected arm (right), n (%)	46 (43)	19 (46)	20 (44)	7 (37)
Affected side (dominant), n (%)	46 (43)	18 (44)	21 (46)	7 (37)
Stroke type, n (%)				
Ischemic	88 (83)	39 (95)	35 (76)	14 (74)
Hemorrhagic	18 (17)	2 (5)	11 (24)	5 (26)
Stroke severity at onset (NIHSS), median (min-max)	7 (0–24)	3 (0–4)	8 (5–14)	18 (15–24)
Motor function at onset (FMA-UE), median (quartiles)	34 (4–56)	56 (47–61)	14 (4–41)	4 (4–9)
Sensory function at onset (FMA-UE), median (quartiles)	11 (11–12)	12 (11–12)	7 (1–12)	0 (0–1)
Grip strength at onset in % of normative values, mean (SD)	38 (41)	59 (35)	28 (41)	16 (38)
Individuals in rehabilitation, n/n of time point (%)				
Day 3	106/106 (100)	41/41 (100)	46/46 (100)	19/19 (100)
Day 10	96/106 (91)	34/41 (83)	44/46 (93)	18/19 (95)
Week 4	80/100 (80)	23/39 (59)	40/43 (59)	17/18 (94)
Month 3	55/90 (61)	19/37 (51)	23/39 (60)	13/18 (93)
Month 6	46/82 (56)	13/31 (42)	22/37 (40)	11/14 (79)
Month 12	29/77 (38)	6/29 (21)	14/35 (40)	9/13 (69)

Abbreviations: *NIHSS* National Institutes of Health Stroke Scale (score range 0–42); *Mild* < 5 points; *Moderate* 5–14 points; *Severe* > 14 points according to the NIHSS; *FMA-UE* Fugl-Meyer Assessment for Upper Extremity (motor score range 0–66; sensory score range 0–12)

The number of participants that completed ABILHAND in the six different time-points varied (Table 3). The main reasons for missing values at early time-points were tiredness and inability to answer the questions adequately (11 and 7 participants at 3 and 10 days, respectively). The drop-out at follow-up time-points was mainly due to decline of the study (16 at 12 months), death (6 at 12 months) and new stroke (7 at 12 months).

**Table 3:** Self-perceived manual ability (ABILHAND logit score) in 6 time-points after stroke

Group/subgroup		Day 3	Day 10	Week 4	Month 3	Month 6	Month 12
**Total group**	Mean	−0.81	0.09	1.45	2.57	2.51	2.91
	SD	3.02	2.75	2.81	2.73	2.69	2.83
	n	95	99	97	88	82	77
**Mild**	Mean	0.41	1.54	2.84	3.51	3.44	3.80
	SD	2.79	2.13	2.19	2.46	2.40	2.34
	n	41	39	39	36	31	30
**Moderate**	Mean	−1.09	−0.39	0.78	2.34	2.35	3.10
	SD	2.57	2.53	2.79	2.69	2.68	2.49
	n	39	43	43	39	37	34
**Severe**	Mean	−3.42	−1.99	−0.23	0.65	0.90	0.37
	SD	3.04	2.89	2.85	2.60	2.60	3.38
	n	15	17	15	13	14	13

Abbreviations: *Mild* < 5 points; *Moderate* 5–14 points; *Severe* > 14 points according to the NIHSS

### Changes of self-perceived manual ability over time

Analyses of repeated measures showed a significant fixed effect of time on self-perceived manual ability over the first year after stroke in the total group and the subgroups (Table 4 and Table 5). No random effects were found. The mean expected change for the total group over the first year after stroke was 0.26 logits per month from a mean expected baseline logits of 0.46. The expected increase was 0.22 logits per month in the subgroup of mild stroke severity, 0.31 in the subgroup of moderate stroke severity and 0.24 in in the subgroup of severe stroke severity. Individuals with mild stroke severity had higher expected mean baseline logits (1.72) than individuals with moderate (0.03) and severe strokes (− 1.56).

**Table 4:** Effect of time and cofactor age on self-perceived manual ability (ABILHAND logit score) in total group

	**Unadjusted model**	**Adjusted model**
Parameter	Estimate 95% CI p-value	Estimate 95% CI *p*-value
Intercept	0.46 0.14; 0.79 0.005	5.63 3.38; 7.88 < 0.001
Time (month)	0.26 0.20; 0.32 < 0.001	0.24 0.21; 0.28 < 0.001
Age (year)	–	−0.06 −0.09; −0.02 0.001
Stroke severity (NIHSS-score)	–	−0.19 − 0.26; − 0.12 < 0.001

Abbreviations: *CI* Confidence interval, *NIHSS* National Institutes of Health Stroke Scale

**Table 5:** Effect of time and cofactor age on self-perceived manual ability (ABILHAND logit score) in three stroke severity subgroups

Parameter	Mild		Moderate		Severe	
	Unadjusted model	Adjusted model	Unadjusted model	Adjusted model	Unadjusted model	Adjusted model
	Estimate 95% CI p-value	Estimate 95% CI *p*-value	Estimate 95% CI p-value	Estimate 95% CI p-value	Estimate 95% CI p-value	Estimate 95% CI p-value
Intercept	1.72 1.28; 2.15 < 0.001	3.63 0.68; 6.57 0.017	0.03 −0.43; 0.49 0.895	4.47 0.44; 8.50 0.031	−1.56 −2,41; − 0.71 < 0.001	0.16 −6.39; 6.71 0.960
Time (month)	0.22 0.14; 0.31 < 0.001	0.62 0.33; 0.90 < 0.001	0.31 0.22; 0.40 < 0.001	0.28 0.22; 0.33 < 0.001	0.24 0.09; 0.40 0.003	0.75 0.29; 1.22 0.002
Age (year)	–	−0.028 − 0.07; 0.02 0.197	–	−0.066 − 0.12; − 0.01 0.025	–	−0.025 − 0.12; 0.07 0.569
Interaction effect of time and age	–	− 0.006 − 0.01; 0.00 0.006	–	–	–	− 0.008 − 0.02; 0.00 0.018

Abbreviations: *CI* Confidence interval, *NIHSS *National Institutes of Health Stroke Scale, *Mild* <5 points; *Moderate* 5–14 points; *Severe* > 14 points according to the NIHSS

The adjusted model for the total group included a significant fixed effect of time and cofactors age and stroke severity assessed by NIHSS-score (Table 4). In this model the expected increase per month was 0.24 logits during the first year after stroke although this increase was negatively influenced by age (− 0.06 logit per year) and stroke severity (− 0.19 per point in NIHSS-score). Cofactors gender, living condition and affected hand did not influence the model significantly.

The adjusted model for the subgroup with moderate stroke severity (Table 5) included a fixed effect of time (0.28 logits per month) which was negatively adjusted by age (− 0.07 per year). The models for the mild and severe subgroups, in addition to a significant effect of time also included an interaction effect of time and age, i.e. the effect of time varied in different age groups. The interaction effect indicated that the older a person was the lesser was the effect of time.

### Changes of self-perceived manual ability between different time-points

**Fig. 2:**
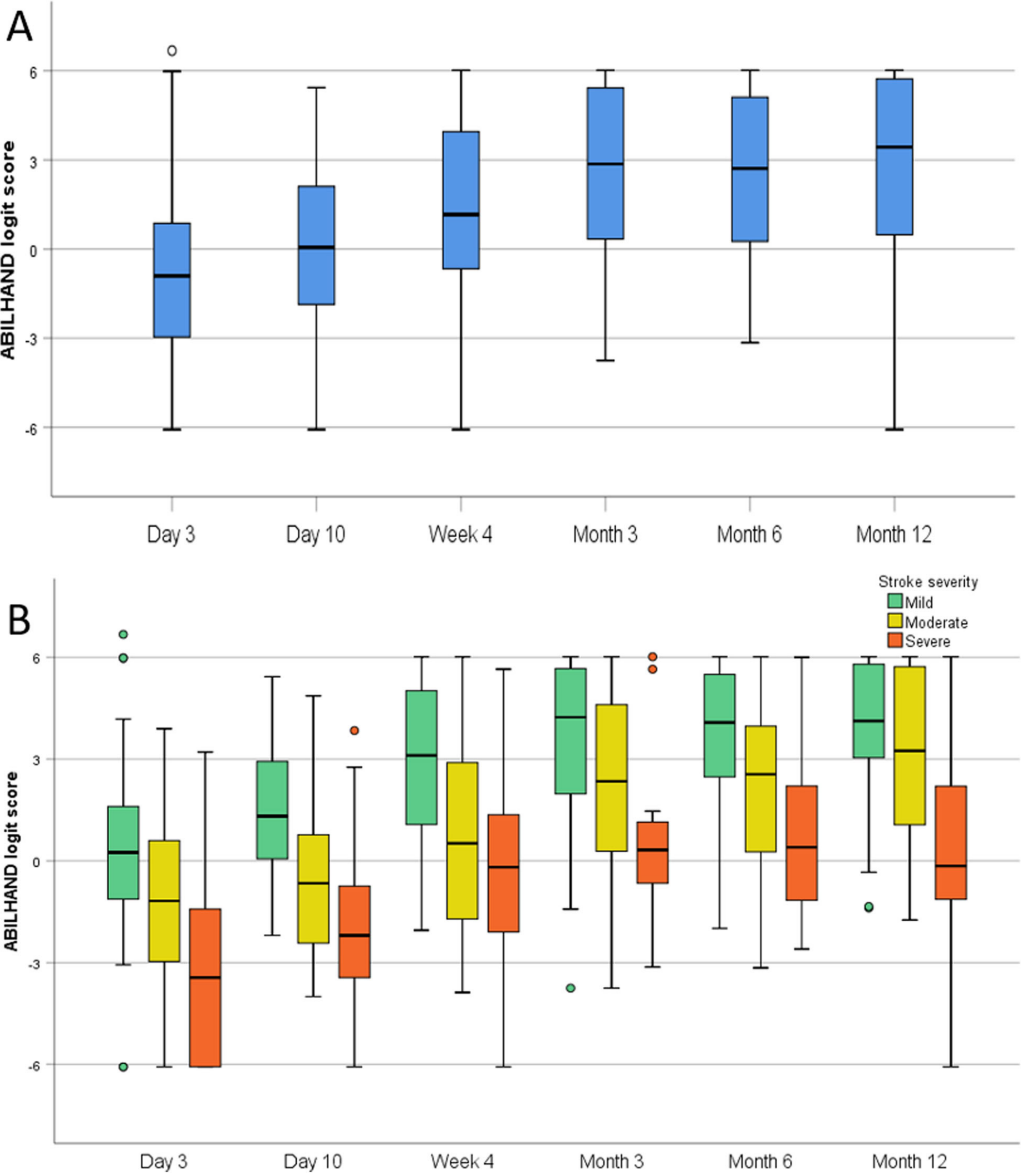
Boxplots of self-perceived manual ability (ABILHAND logit score) in six time points after stroke for total group (**a**) and stroke severity subgroups (**b**)

Figure 2 visually displays how self-perceived manual ability (logit score) changes in six time-points over the first year post-stroke for the total group (a) and subgroups (b). Significant increases in the total group were seen between ‘Day 3’ and all other time-points from ‘Week 4’ and forward, between ‘Day 10 and all time-points from Week 4’ and forward as well as between ‘Week 4’ and the time-points ‘Month 6 and 12’ (see Table 6). No significant change was detected beyond 3 months post-stroke.

**Table 6:** Pairwise comparisons of differences in self-perceived manual ability (ABILHAND logit score) in 6 time points after stroke for total group and subgroups

Pair of time-points	Total group *p*-value (n)	Mild *p*-value (n)	Moderate *p*-value (n)	Severe p-value (n)
Day 3 - Day 10	0.981 (93)	1.000 (39)	1.000 (39)	1.000 (15)
Day 3 - Week 4	< 0.001 (87)	< 0.001 (39)	0.015 (36)	0.075 (12)
Day 3 - Month 3	< 0.001 (80)	< 0.001 (36)	< 0.001 (33)	0.002 (11)
Day 3 - Month 6	< 0.001 (73)	< 0.001 (31)	< 0.001 (30)	< 0.001 (12)
Day 3 - Month 12	< 0.001 (70)	< 0.001 (30)	< 0.001 (30)	0.005 (10)
Day 10 - Week 4	< 0.001 (91)	0.036 (37)	0.102 (40)	0.959 (14)
Day 10 - Month 3	< 0.001 (83)	< 0.001 (34)	< 0.001 (36)	0.075 (13)
Day 10 - Month 6	< 0.001 (78)	< 0.001 (30)	< 0.001 (34)	0.012 (14)
Day 10 - Month 12	< 0.001 (73)	< 0.001 (29)	< 0.001 (32)	0.128 (12)
Week 4 - Month 3	0.122 (87)	1.000 (36)	0.520 (38)	1.000 (13)
Week 4 - Month 6	0.005 (80)	1.000 (31)	0.031 (36)	1.000 (13)
Week 4 - Month 12	< 0.001 (75)	0.251 (30)	0.001 (33)	1.000 (12)
Month 3 - Month 6	1.000 (78)	1.000 (30)	1.000 (35)	1.000 (13)
Month 3 - Month 12	1.000 (73)	1.000 (29)	1.000 (32)	1.000 (12)
Month 6 - Month 12	1.000 (74)	1.000 (30)	1.000 (32)	1.000 (12)

Significant values have been adjusted for the Bonferroni correction for multiple tests

## Discussion

The current study showed that self-perceived manual ability increased over time the first year after stroke both in the total group and the stroke severity subgroups. The effect of time was modulated both by age and stroke severity in the total group. In the subgroup of moderate stroke severity, similarly to the entire group, age had a significant effect in the model. In the subgroups of mild and severe stroke an interaction effect between time and age was found, which means that the increase in logits was different in different age groups. The interaction effect indicated that the effect of time was reduced in those who were older. Further analyses of change in perceived manual ability between time-points showed that most of the improvements occurred early, predominantly within the first three months after stroke.

Time was the factor that had largest effect on manual ability in the total group and in the subgroups. This is in line with previous research that has shown that time is an independent factor that reflects spontaneous recovery of body functions and activities after stroke [28]. Even though manual ability varied between individuals at baseline in the present study the changes over time were independent from these differences. This has also been found in a previous study where self-perceived manual ability (ABILHAND logits) after a targeted rehabilitation intervention between subacute and chronic phase showed same patterns of change over time in subgroups with varying stroke severity [18].

Recovery of the upper extremity body functions and activity capacity after stroke will occur over time and initial severity of motor impairment is an important predictive factor for outcome [28–31]. Changes in impairments during the first weeks after stroke will result in concomitant improvements in activity capacity [32] and recovery over the first months will also be reflected in self-perceived manual ability [33]. However, besides the actual return of manual ability the perception may be affected by other factors such as expectations on recovery and functioning, ability to compensate for disability and adapt to new circumstances after stroke [34]. Previous research has shown that changes in objective and observed measures do not always correspond to self-perceived measures [18, 35, 36]. Measuring both objective, observed and perceived manual ability is therefore important when following recovery of the upper extremity after stroke.

In the adjusted model the effect of time was to a certain degree modulated by stroke severity and age at onset. Accordingly, in older people and in case of more severe stroke the effect of time will be lower compared to younger people and those with less severe stroke. This is in line with previous studies that have found that age and initial stroke severity are important predictive factors for outcome of activities of daily living post-stroke [32, 37, 38]. Stroke severity and age both are associated to increased comorbidity and lower physical and cognitive function [37, 38]. The results from the current study showed that these factors also had an impact on the perceived manual ability after stroke.

In the current study, several other cofactors were tested in the models. In line with previous research [37], being male or female did not have significant effect on increased perceived manual ability in the current study. Having the dominant arm affected can be considered as a larger disability compared to the non-affected arm. However, having the dominant hand affected did not impact the change in perceived manual ability. A possible explanation could be that, an affected dominant hand would be forced into activities to larger degree than a non-dominant and this might result in larger training dose which might lead to increased regained functioning [39]. ABILHAND is also designed to measure the bimanual ability, which might mask the more subtle differences in hand dominance.

In previous research it has been shown that motor recovery is almost completed after four to 10 weeks [28]. The time course is characterized by larger improvements during the first weeks [28, 29, 31, 40]. Also, recovery of body functions will precede the recovery of activities and therefore reach a plateau phase sooner (about two weeks) [32]. As manual activity performance relies on learning new motor processes and compensatory skills and on adaptation to new conditions in daily life, recovery can continue over a longer time. In fact, the current study showed that changes in perceived manual ability improved from stroke onset to one year after stroke. However, most increase in logits was seen early on and after three months the increase levelled out. One reason for this might be due to ceiling effects in ABILHAND in the mild and moderate group. There are some suggestions from previous studies to extend the range of measurement in ABILHAND by adding more difficult items to reduce this ceiling effect [25, 41]. The rehabilitation interventions are also more concentrated during the first months after stroke, which might influence the perceived manual ability. Interestingly, in rehabilitation wards individuals tend also to perceive manual ability more positively, in contrast to ordinary daily life circumstances when living at home [18]. When returning home, one might become more aware of the problems in manual activities in real life environments, which might lead to lower ratings. Non-use phenomena and peripheral biomechanical changes of the upper extremity might also have negative effects on manual performance in chronic stages of stroke [10].

The improvement in perceived manual ability for the subgroup of severe strokes levelled out earlier than in the mild and moderate group. Previous research has found that about 60% of individuals with severe upper extremity impairment show limited recovery of manual ability [32]. In addition, the recovery after a severe stroke does not follow the same expected pattern as in moderate and mild strokes and the prognosis is less predictable [15]. The present study showed that variations between measurements and between individuals were larger in the group with severe stroke compared to mild and moderate.

Living with the consequences of stroke often means a lifelong adaptation to the disability. In this process, the individual’s own perception of difficulties plays a central role, and should guide training and development of strategies to manage meaningful daily activities. Therefore, self-perceived outcome measures are important as they provide information on what matters to the individual that might not always be captured in assessments of function and activity capacity. However, it has been argued that perceived performance in daily activities is difficult to rate early after stroke since the activities have not yet been tried [32]. ABILHAND has, however, the advantage that the logit score is based on the tasks that actually have been performed in real life [11, 27], which makes it an attractive measure to use even early after stroke.

The present study does have some limitations that should be regarded when interpreting the results. Different aspects of functioning e.g. cognitive function, alertness, mood, fatigue and social habits might influence the self-reported outcomes. In the current study, when the participant was not able to adequately answer the questions of the ABILHAND, the test was recorded as missing. Also, patients with comorbidities that possibly could influence the upper extremity functioning were excluded from the study. Nevertheless, other factors, not included in the current study, such as, mild cognitive deficit, general physical function or amount of rehabilitation received might have influenced the results of the self-reported manual ability. The number of participants varied between time-points partly due to limited ability to respond to the questions of the ABILHAND early after stroke, and partly due to the general drop-out from the study over time. However, the mixed model of repeated measurements was chosen as it handles missing values. Even if the sample size in the present study was sufficiently large to evaluate the impact of time and cofactors, subgroups analyses must be interpreted with care. Few significant differences between time-points were found in the subgroup of severe strokes which might have been due to the small sample. Nevertheless, a strength of the current study is the unselected sample which also included severe stroke as this group often is omitted and therefore less investigated in research. This also enhances clinical generalizability of results. Furthermore, well-defined and recommended follow-up time-points after stroke were used [6].

Increased knowledge about changes over time for individuals with stroke and in subgroups of stroke is important and needed. Results from the current study contribute to an improved understanding of perceived manual ability during the first year after stroke. Future studies in larger samples could further increase knowledge regarding longitudinal change in different subgroups of stroke severity.

## Conclusion

In conclusion, the present study showed that the self-perceived manual ability increased over the course of the first year after stroke. This increase was however, to some degree influenced negatively by older age and more severe stroke. Most of the improvements in manual ability occurred early, predominantly within the first three months after stroke.

## Data Availability

Interested researchers may submit requests for data to the authors (contact ks.sunnerhagen@neuro.gu.se). According to the Swedish regulation (http://www.epn.se/en/start/regulations/), the permission to use data is only for what has been applied for and then approved by the ethical board.

## Abbreviations

NIHSS: National Institutes of Health Stroke Scale
FMA-UE: The Fugl-Meyer Assessment for Upper Extremity
ARAT: Action Research Arm Test
SALGOT: Stroke Arm Longitudinal Study at the University of Gothenburg
